# The role of interactions within the family in the psychological well-being of postmenopausal women in shiraz (Iran)

**DOI:** 10.1192/j.eurpsy.2023.1357

**Published:** 2023-07-19

**Authors:** M. Hosseini

**Affiliations:** Social Sciences, University of Bojnord, Bojnord, Iran, Islamic Republic Of

## Abstract

**Introduction:**

As one of the most basic social institutions, the family plays an important role in different periods of people’s lives. One of these periods, which are usually associated with a crisis and change in the physical and mental conditions of women, is menopause, which requires the provision of suitable conditions for transition, especially in the family.

**Objectives:**

the purpose of this research is to study the role of interactions within the family in the psychological well-being of postmenopausal women.

**Methods:**

To achieve this goal, qualitative content analysis method has been used. The main question of this research is how do family interactions play a role in the life of postmenopausal women? The participants of this research were 15 menopausal women aged 45 to 60 years old in Shiraz (Iran) who were selected using the purposeful sampling method. Data were collected, coded and analyzed using in-depth and semi-structured interviews. In order to achieve the accuracy and reliability of the data, Guba and Lincoln reliability criteria have been used.

**Results:**

The analysis of the interviews of the participants in this research led to the extraction of 6 subcategories included, “mental rumination in sexual relations”, “changes in marital intimacy”, “insufficient interactions in the family”, “resistant normative femininity and motherhood”, “fear of aging”, “female shame and taboo”, and the two main categories included “lack of awareness as a relationship parasite” and “destructive resistance”.

**Conclusions:**

The findings of the research indicate that the family members’ lack of knowledge about this period and the prevailing culture of female shame and the taboo of women’s bodies make the family unable to provide the necessary support to menopausal women. On the other hand, keeping menopause a secret and emphasizing on maintaining pre-menopausal conditions by women is a destructive resistance that ultimately leads to psychological damage to them. Therefore, it is suggested that in addition to holding training programs for women in order to enter and face this period properly, trainings should also be considered for other family members and especially husbands (men). It seems that family members can play an effective role in various stages of menopause, including preparation and psychological adaptation of women by receiving correct training.

**Disclosure of Interest:**

None Declared

